# Assessing body position through experimental cremation: A pilot study using colorimetry and FTIR-ATR analyses

**DOI:** 10.1371/journal.pone.0351767

**Published:** 2026-06-15

**Authors:** Paula Becerra Fuello, Javier Lescure, Aaron Lackinger, María Sedeño Ráez, Jesús Gámiz Caro, Gonzalo Aranda Jiménez

**Affiliations:** 1 Department of Prehistory and Archaeology, University of Granada, Granada, Spain; 2 Physical Anthropology Unit, Department of Biodiversity, Ecology and Evolution, Faculty of Biological Sciences, Universidad Complutense de Madrid, Madrid, Spain; 3 University Institute for Research in Iberian Archaeology, University of Jaén, Jaén, Spain; Universidad de Sevilla, SPAIN

## Abstract

This pilot study evaluates the feasibility and limitations of a multi-proxy approach for identifying potential indicators of horizontal positioning in cremated heads from archaeological, and to a lesser extent, forensic contexts. Two outdoor experimental cremations using fleshed and dry pig crania were conducted to evaluate the influence of pre-burning condition, vertical placement within the pyre and pyre dynamics on the expression of lateralised burning patterns. Combining macroscopic observations, fragmentation, colorimetric and Fourier-Transform Infrared Spectroscopy in Attenuated Total Reflectance (FTIR-ATR) mode, our preliminary observations suggest that lateralised differences in thermal exposure may be detectable under certain conditions. Significant differences (p-value < 0.005) were found between direct contact areas and indirect contact areas, with direct contact regions exhibiting lighter colouration and greater calcination. Higher crystallinity (IRSF) was also noted in direct contact areas, especially for the head placed on top of the pyre (Head 1). Inner surfaces consistently retained darker hues, indicating tissue shielding effects. Fleshed and dry elements also behaved differently: the dry cranium was less fragmented and with a more homogeneous colouration. As a small-scale experiment involving heterogeneous specimens and variable pyre dynamics, this study does not attempt to reconstruct body position but instead identifies methodological variables with potential for future replicated research. These preliminary results support the value of combining multi-proxy analyses such as colorimetry and FTIR-ATR for detecting specific burning patterns and inferring body position in cremation studies and provide a starting point for the refinement of methodology in future experimental works.

## Introduction

Understanding the original position of bodies in cremations, either accidental or intentional, remains one of the underexplored but critical aspects in archaeological and potentially in forensic contexts. This particular aspect of the pre-burning condition has often been overshadowed by other variables, mainly the presence of soft tissues, whether the bodies were fleshed or dry, through the analysis of heat-induced fractures, the presence of warping or colour patterns (e.g., [1–6]) as well as pyre configuration, i.e., closed vs open environment [7], or fuel type [8–10]. More recently, the use of FTIR-ATR, which allows the analysis of changes in the chemical structure of bone during burning processes through infrared indices, together with isotopic approaches, has improved our understanding of some of the previous variables, as well as of other fire-related conditions (e.g., [11–18]).

The reconstruction of pre-burning conditions (e.g., original moisture, wet vs. dry state, completeness and position of the individual) through experimental analyses is highly relevant for interpreting funerary behaviour in archaeological contexts, as well as potentially for forensic interpretations. Experimental cremations allow us to investigate how bodies —and more precisely bones— react to different fire environments and pre-burning conditions. The number of publications on experimental outdoor cremations has increased rapidly in recent decades (e.g., [7,11–14], see Thompson and Nannetti [19] for an overview of the current trends). Many of these experiments have aimed to replicate ancient practices under controlled outdoor conditions to investigate decomposition states, multi-sensory experiences, and the burning process [7,11–14,20–26,27,28].

Despite these advanced experiments, few attempts have been made to assess differential burning on different sides of anatomical elements, or to correlate side-specific discolouration with body orientation. Recent experimental work has successfully explored vertical body position within the pyre, either top, middle, or bottom of the structure, using isotope analyses and FTIR-ATR [11], while other studies have approached body position through side-specific burning patterns on a macroscopic level [3,6,29–36]. Nonetheless, robust methodologies specifically designed to evaluate lateralised burning patterns in bones, combining macroscopic evaluation with colorimetry or FTIR-ATR spectroscopy, are still lacking.

This study aims to evaluate the feasibility of a multiproxy approach combining macroscopic observation, colorimetry, and FTIR-ATR spectroscopy, to explore differences between sides with heat-induced alterations through the comparison of Direct Contact Areas (DCA) and Indirect Contact Areas (ICA) with the flames, while considering other factors that can modify this lateralised pattern, such as the pre-burning condition, vertical placement, and pyre dynamics that contribute to heterogeneous conditions. While the broader goal of this research is to contribute to methodologies relevant for body-position research in cremation contexts, the present work constitutes a pilot study focused on identifying which factors affect the potential detection of differential burning patterns. It also examines how variation in specimens and pyre dynamics influences these signals and presents some methodological considerations for future, larger scale, replicated experiments. To ascertain whether such distinctions can be observed in terms of side (left/right) despite different vertical positions, two experimental cremations were conducted at the University of Granada. The resulting remains were analysed using colorimetry and FTIR-ATR spectroscopy to evaluate structural and chemical transformations in the bone.

## Materials and methods

### Experimental design

The two experimental outdoor cremation pyres were built in the same place at the University of Granada facilities (coordinates UTM datum WGS 84: 30S 446825.08E 4116734.43N) during different months (October and December 2024). On one of these experimental pyres, the pig crania were placed on the top, and on the second, at the bottom. Each cremation event was observed by more than ten participants, who completed standardised forms (Form S1 File) documenting the qualitative and observable changes in the burning process, while the temperature was measured with a single pyrometer. The high number of observers improved the recording of small changes in the pyre structure and in the state of the samples. The forms completed by the participants were used exclusively for the qualitative observations included in Supplementary Information S1 File for each pyre. All participants provided informed verbal consent during the experiments, and no personal data were collected or used in this study; therefore, all observations were analysed anonymously. The forms were completed on the days of the experiments (11 October 2024 and 13 December 2024). The selection of fleshed pig heads (n = 2) and one dry pig cranium (n = 1) as animal carcasses was based on their availability as commercial offal, meaning that no animals were slaughtered specifically for research purposes.

### Sample selection and pyre construction

The selection of adult fleshed pig heads (n = 2) and one dry pig cranium (n = 1) as animal carcasses was based on their availability as commercial offal, meaning that no animals were slaughtered specifically for research, together with the similarities between pigs (*Sus scrofa*) and the human body [7,37,38]; and existing evidence showing that different mammalian species will have similar patterns of thermal alteration [39–41]. Both fleshed pig heads were purchased from a local butcher, and the remaining body parts were used for commercial meat consumption. The head burned on Pyre 1 had part of the cheek muscles from both sides removed by the butcher prior to the experiment for commercial purposes (see Fig 2).

Half of a dry pig cranium (sagittally sectioned) was also included in the first experiment to assess differences in fragmentation and colouration between fleshed and dry remains and serving as a control sample to determine if DCA/ICA colour differences could also occur in dry, fragmented bones. This cranium was recovered from an outdoor environment in the province of Almería (approximate UTM datum WGS 84: 30N 535300.00E 4112900.00N). It exhibited signs of prolonged sun exposure and complete desiccation, with no remaining soft tissues. Although the exact post-mortem interval could not be established, its taphonomic condition is consistent with long-term surface exposure, presenting some weathering. Because the dry cranium was introduced into the pyre later than the fleshed head in the burning sequence, its thermal exposure also differed in timing and combustion dynamics (see S6 File). The dry cranium was introduced later in Pyre 1 because defleshed skeletal elements are expected to combust and reach calcination more rapidly than fleshed remains due to the absence of shielding soft tissues and their lower moisture content. The delayed placement was therefore intended to reduce excessive thermal alteration and to allow a more comparable exposure time between the dry and fleshed specimens under the same pyre conditions. This constraint is inherent to the pilot nature of the experiment and should be considered when comparing the results between fleshed and dry bone.

Both pyres were built using olive wood, branch wood, pine leaves and pinecones. Olive wood was chosen due to its combustion stability [7], its widespread availability in southern Iberia and its traditional use in the region. Moreover, archaeological evidence of olive wood charcoal has been identified in cremation contexts dating to the Late Neolithic and Chalcolithic periods in southern Iberia [8]. In addition to wood, a minimal amount of pine-based fire starters was used on the first pyre, together with pinecones to facilitate ignition. In contrast, the second pyre incorporated at least ten small fire starter fragments.

**Fig 1:**
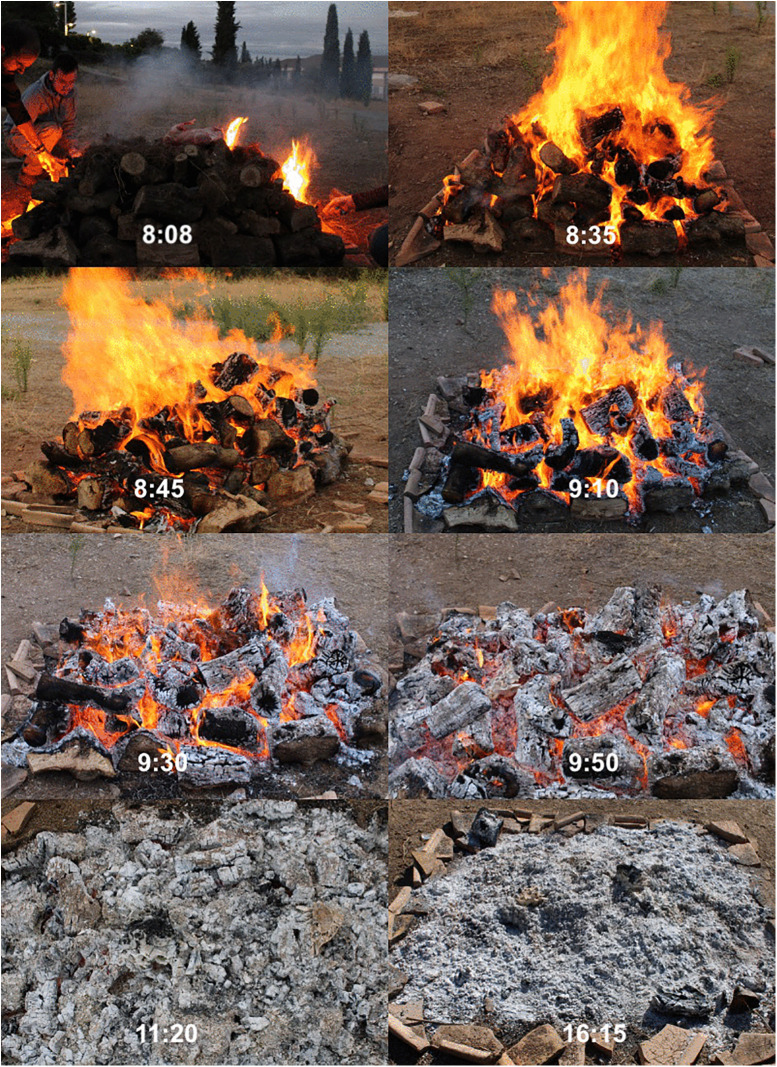
Burning event and changes in Pyre 1 structure through the experiment.

- **Pyre 1:** Outdoor Experimental Cremation (11 October 2024). This pyre had a rectangular structure with four layers of olive wood logs (total weight: 196.5 kg), measuring 1.4 m (length) × 1 m (width) × 0.5 m (height). The logs were arranged horizontally with spacing for airflow. The weather conditions were cloudy with intermittent rain, starting at 11°C (humidity: 67–72%; wind: ~ 5.8 km/h, easterly), peaking at 21°C. The fleshed pig head weighed 5.05 kg and the dry cranium 120 g. The fleshed pig head was deposited on its left side. The experiment lasted 8 h. All changes in the pyre are shown in Fig 1 and described in detail in Supplementary Information S6 File.

**Fig 2:**
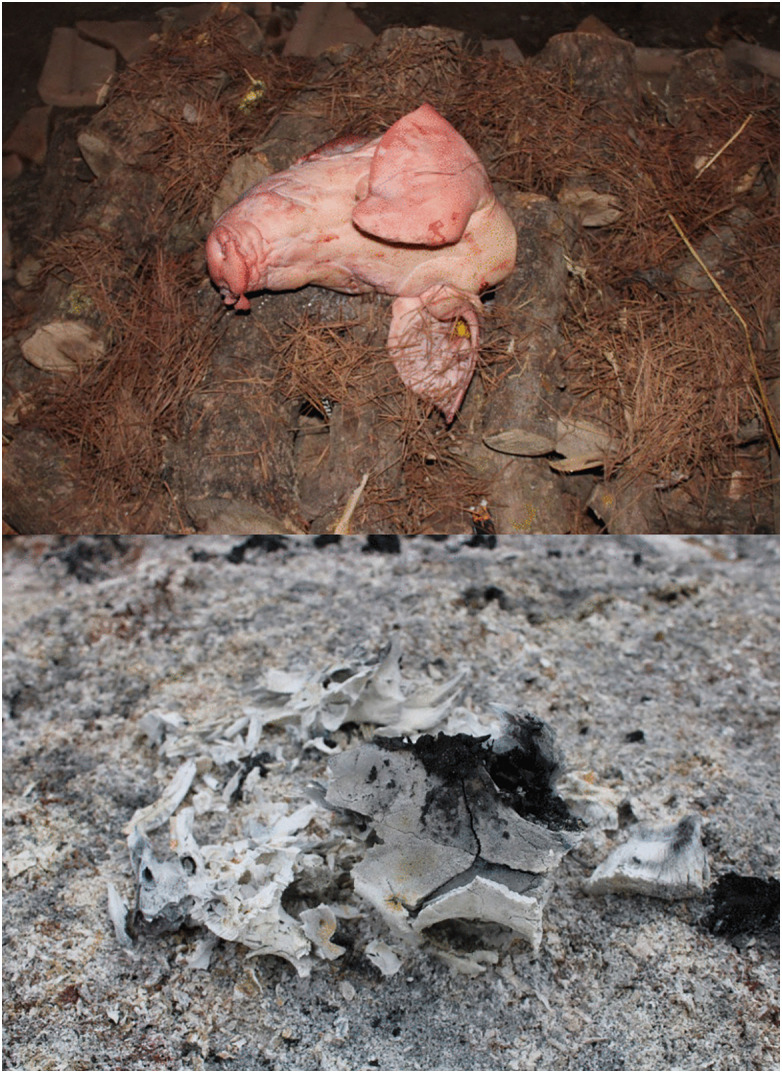
Fleshed pig head (Head 1) before ignition (above) and after cremation (below).

The bone remains were recovered on 14 October, three days after the cremation and after some additional rainfall. A photographic record of the outcome of Pyre 1 was taken prior to bone recovery in order to create a 3D model to ascertain the differences in dispersion between fleshed and dry bones (see S2 File; also published on the open website Sketchfab: https://skfb.ly/pyAHQ). Fig 2 also shows the pre-burning condition of the pig head before ignition and the result of the pyre on 14 October, three days after the fire event.

**Fig 3:**
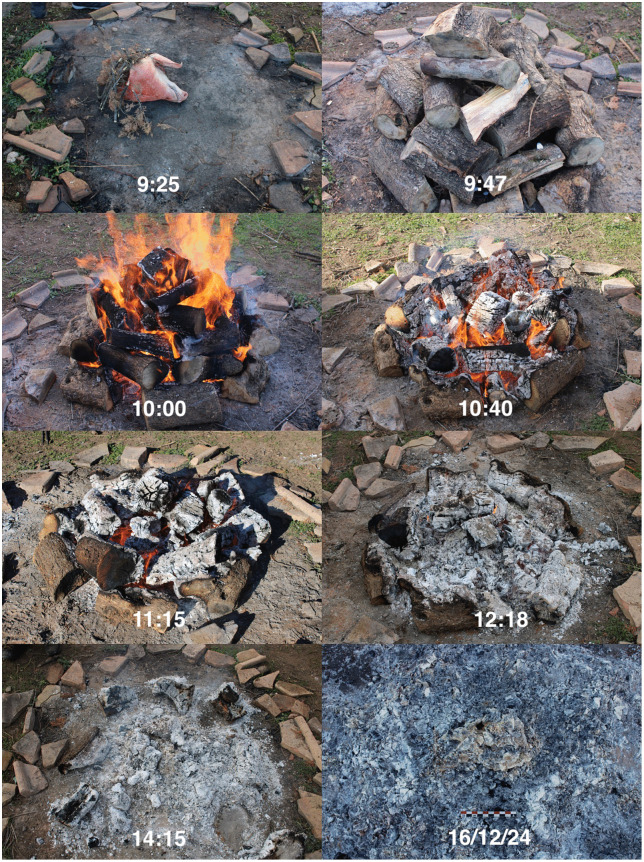
Burning event and changes in Pyre 2 structure through the experiment and the day of collection (three days later).

- **Pyre 2**: Outdoor Experimental Cremation (13 December 2024). This circular pyre included 76.74 kg of olive wood and pine elements. The structure had a maximum diameter of 90 cm and height of ~50 cm. Ambient conditions were cooler and more humid than with Pyre 1 (6°C, 78% humidity, 2 km/h E wind). Combustion was faster and more intense than Pyre 1, concluding once all the wood had been fully combusted. The fleshed pig head used for this experiment weighed 5.95 kg, it was deposited on its left side, following the same positioning used in Pyre 1, and no other fleshed or dry elements were used in this experimental cremation. The position, structure and events are also depicted in Fig 3. The experiment lasted around 5 hours, and all the details are described on S6 File.

These heterogeneous combustion conditions, inherent to the exploratory and pilot nature of the experiment, allow us to evaluate how pyre structure and environmental differences affect the outcomes of the analytical methods applied. Furthermore, the differences in the amount of wood between pyres and the circular disposition of Pyre 2 allowed us to assess whether pyre structure and fuel quantity affected the calcination level, and the potential lateralized pattern observed in the pig heads. Nevertheless, these variables should be considered when comparing the results between both pyres.

### Temperature monitoring

Surface temperatures were monitored throughout the cremation process using a BTMETER BT-1800 dual infrared laser thermometer/pyrometer featuring a 50:1 distance-to-spot ratio and adjustable emissivity (0.1 to 1.0). Temperature precision was ± 3% or ±3°C for –50°C to 0°C, and ±2% or ±2°C for 0°C to 1000°C. Temperature readings were taken placing the infrared dots on both the fleshed pig head and the dry cranium to compare thermal exposure between the two elements. As thermocouples were not used, temperature data were recorded every 15 minutes until all the fuel had been consumed.

### Macroscopic evaluation

All the recovered bone and dental fragments were sorted by anatomical region: side, colouration and size. Fragments were sorted in size categories: < 5 mm, 5–20 mm, 20–50 mm and >50 mm. The identification rate was higher in those larger than 50 mm. After sorting and identification all fragments were weighed.

Main colour, colour pattern, warping and heat-induced fractures were also recorded on the database, to check the differences between the individuals (see S8 File). Colouration patterns were evaluated on both the outer (exocranial) and inner (endocranial) surfaces. Direct Contact Areas (DCA) and Indirect Contact Areas (ICA) were defined according to the original anatomical orientation of each head prior to burning. The colouration of bones exposed to fire usually changes from yellowish to brown and black, different hues of grey, and finally to chalky white as calcination progresses, depending on the intensity and duration of fire exposure. These colours were recorded (see S8 File) following previous literature [20,42]. Fragments lacking secure anatomical orientation were excluded from DCA/ICA comparisons.

### Colorimetric analysis

The colorimetric analysis was conducted with a PCE-CSM 2 colorimeter (PCE Instruments, Meschede, Germany) The supported colour spaces of the PCE-CSM 2 colorimeter are CIEL*a*b*C*h, CIEL a*b*, and CIEXYZ. The measurements evaluated here were taken in the CIELAB system (L*a*b*) with a measuring aperture of 8 mm. Before measurement, the device was calibrated according to the manufacturer’s instructions, although calibration of black-and-white contrast was also performed every 100 individual measurements. In the CIELAB colour parameters: L* value measures the lightness or brightness of a colour (white = 100, black = 0), a* value indicates colours between green and red (positive + = redness, negative - = greenness), b* value measures yellowness or blueness (positive += yellow, negative - = blueness) [43]. The intersection of these three coordinates gives the value of the colour later evaluated statistically. Colour was evaluated both macroscopically and through a colorimeter. For the colorimetric analysis 12 samples were taken from each bone fragment: 3 from the lightest colour of the outer part of the skull, 3 from the darkest colour of the outer part, 3 from the lightest colour of the inner part of the skull, and 3 from the darkest colour of the inner part of the skull. With these measurements we were able to evaluate not just the colour range but also the location of the measurement in each bone fragment studied.

The colorimeter was only used on those skeletal parts with a 100% confidence of anatomical identification and siding (n = 27), yielding a total of 153 colorimetric measurements for Head 1, 93 for Head 2 and 24 for the dry cranium.

Furthermore, in those same measurements, L* and B* coordinates were used to estimate burning temperature ranges using the decision model proposed by Krap et al. [41,44]. This model groups LAB values into seven temperature clusters based on experimentally derived L*–B* thresholds and was used to assign each measurement to the closest temperature category. These estimations provided approximate minimum and maximum exposure ranges for each sampled surface (DCA/ICA), allowing comparison with the infrared pyrometer data.

### Fourier-Transform Infrared spectroscopy in Attenuated Total Reflectance (ATR) mode analysis (FTIR-ATR)

The analysed bone fragments were taken from left and right sides of the parietal and temporal bones of Head 1 and Head 2, with a total of 4 samples per individual (left parietal, left temporal, right parietal and right temporal). In the case of the dry cranium, one sample was taken from the splanchnocranium.

The samples were pretreated and analysed in accordance with the methods described by Stamataki et al. [12]. Mechanical cleaning of the internal and external surfaces of the fragments was carried out using a drill to remove sediment and trabecular bone. Pre-treatment continued with the chemical cleaning of each sample. First, each sample was rinsed three times in MilliQ water in an ultrasonic bath for 10 minutes each time. They were then cleaned in 10 ml of 1M acetic acid and subjected to a single ultrasound bath for 10 minutes. Finally, the rinsing process was repeated in MilliQ water in an ultrasonic bath for 10 minutes each time. The fragments were dried in ceramic pots that were placed in an oven at 50°C for a minimum of 12 hours.

Once this process had been completed, the samples were ground with an agate mortar and pestle and sieved according to the protocol described by Kontopoulos et al. [37]. A column of 50-μm and 25-μm steel mesh sieves was used to sieve the bone powder, and the spectrometer analysis was conducted using exclusively the 50-μm-25-μm particle size.

The FTIR-ATR analyses were conducted at the Scientific and Technical Instrumentation Centre (CICT) of the University of Jaén. The spectrometer model used was the Bruker Vertex 70 (without vacuum) with a spectral range of 4000–400 cm^−1^ and a spectral resolution of 4 cm^−1^. The scan configuration was set to 32 scans in Absorbance Mode. For each sample, 2–3 milligrams of bone powder were used. After each measurement, the crystal plate and anvil of the pressure applicator were meticulously cleansed with ethanol. Prior to the analysis of each sample, the background signal was measured. Three measurements were taken for each sample, and the indices were calculated using the average and standard deviation of these three measurements.

The spectra were processed using OPUS 7.6 software, and baseline correction was performed manually according to the protocol outlined by Stamataki et al. [12]. The indices considered in this study were C/C, IRSF, C/P, P/P, BPI, OH/P, CN/P and Am/P: Infrared Splitting Factor (IRSF), which provides information on the crystallinity of hydroxyapatite [12,15]; carbonyl-to-carbonate ratio (C/C), which evaluates the amount of carbonyl groups, as calcined bones completely lose these compounds [15,45]; type B carbonate-to-phosphate ratio (C/P), which compares carbonate and phosphate content, with lower carbonate values generally associated with higher degrees of calcination [29,45]; phosphate-to-phosphate ratio (P/P), which compares two phosphate absorbance bands [12], where the peak at 560 cm ⁻ ¹ becomes more pronounced with increasing temperature [45]; B carbonate-to-phosphate index (BPI), which measures the phosphate peak at 605 cm ⁻ ¹ and decreases with increasing temperature and burning duration [15]; hydroxyl-to-phosphate ratio (OH/P), which evaluates changes in hydroxyl group content in bone apatite [15]; amide-to-phosphate ratio (Am/P), which measures the amount of organic matter and water relative to phosphate content [12]; and cyanamide-to-phosphate ratio (CN/P), associated with reducing combustion conditions and the presence of ammonia, which has been linked to the burning of shrouds or garments [11]. Nevertheless, is important to note that as the FTIR spectrometer was not under vacuum and cyanamide (H_2_CN_2_) presence is not detectable under the CN/P ratio of 0.25 [11,46].

### Statistical analysis

Prior to the actual data analysis, we conducted a review of samples to identify outliers based on the L*a*b values. Given the multivariate nature of the data, we applied the Mahalanobis distance to the multivariate sample mean, which accounts for the covariance structure of the information [47], with an α level of 0.1. This approach provides more accurate outlier detection than univariate methods when the variables are correlated [48].

To test differences between groups, we opted for a permutational MANOVA [48], an analysis of variance using distance matrices that is directly analogous to parametric MANOVA [49,50]. All the statistical workflow, from the outlier filtering to the visual representation, was performed in R [51–54]. This process was carried out to compare DCA and ICA regions and the vertical position inside the pyre (top/bottom). To visualise the colour points, we needed to reduce the dimensionality from three dimensions (L*a*b) to two, so they could be plotted in a scatterplot. For that purpose, we chose a principal coordinate analysis (PCOA), also known as weighted classical multidimensional scaling [55,56]. To test the differences between the two fleshed pig heads in the FTIR-ATR index, we applied the Wilcoxon test, after performing a Shapiro-Wilk normality test, which showed that the values of our sample for the IRSF differed from the normal distribution.

## Results

Before presenting the results of each analysis, it is important to note that the evaluation of lateralised burning patterns in this pilot study was influenced by the aforementioned factors: (1) the pre-burning condition (fleshed vs. dry), (2) the vertical placement within the pyre (top vs. bottom), and (3) pyre dynamics, including fuel distribution, timing, collapse or environmental conditions. The following subsections present the outcomes of each analysis considering those factors.

### Temperature overview

Pyre 1 reached a maximum temperature of 870 °C, measured on the dry cranium 30 minutes after its placement. The average temperatures recorded were 390 °C for the fleshed head and 345 °C for the dry cranium.

Pyre 2 reached a maximum temperature of 953 °C, recorded 25 minutes after ignition, with an average temperature of 644 °C on the head measurement. Detailed temperature profiles and pyre stages are presented in Supplementary Information (S4 and S6 File).

Colorimetric values were also converted to estimated burning ranges using the model of Krap et al. [56] for colorimetric analysis of burnt bone. Estimated temperatures ranged between 0–350 °C (minimum) and 800–900 °C (maximum) in both pig heads, with maximum average values of 739 °C for Head 1 and 736 °C for Head 2 (see full range of [44] temperature categories in S5 File). The highest colorimetric estimates were consistent with the IR pyrometer measurements.

The dry cranium, due to its orangeish coloration, yielded lower estimated values under this model. Also, most DCA surface measurements of the fleshed heads corresponded to L*a*b* values associated with 700–800 °C, as reported by Fredericks et al. [57].

### Mass loss and fragmentation

Mass and fragmentation data were recorded to characterise the extent of thermal alteration prior to colourimetric and FTIR-ATR sampling.

Mass loss evaluation showed that Head 1 (from Pyre 1) (5.05 kg pre-cremation) was reduced to 380.97g post-cremation (92% loss), while Head 2 (5.95 kg) resulted in 359.63g (94% loss). The dry cranium dropped from 120 g to 92.89 g (9% loss), probably due to enamel detachment and the fragmentation and loss of fragments from the nasal bone (Fig 6). There was a difference of 2% more loss in Head 2 than in Head 1, although this difference is not significant nor interpretable in a pilot study of this scale.

Regarding fragmentation, fleshed head from Pyre 1 (Head 1) was very fragmented. A total of 1,959 fragments were recovered: 89% were of less than 20 mm (within that group, 57% were smaller than 5 mm), 8% ranged between 20–50 mm, and only 2% of the fragments measured more than 50 mm.

From the fleshed head from Pyre 2 (Head 2) 3,811 fragments were recovered: 94% of the bone fragments were less than 20 mm (58% smaller than 5 mm), 5% from 20 to 50 mm, and only 1% over 50 mm, reflecting a greater fragmentation pattern than Head 1 (see S8 File for the detailed distribution of fragment counts).

The differences in fragmentation patterns between the two fleshed heads were statistically significant (Pearson’s chi-square test: χ² = 45.117, df = 2, p < 0.001), indicating that Head 2 was more extensively fragmented into smaller-sized pieces. However, direct comparison between heads must be considered with caution given their differing pyre conditions and fuel distributions.

### Macroscopic evaluation

To provide context for colorimetric and FTIR analyses, macroscopic observations were recorded for all recovered cranial fragments from Heads 1 and 2 and from the dry cranium.

**Fig 4:**
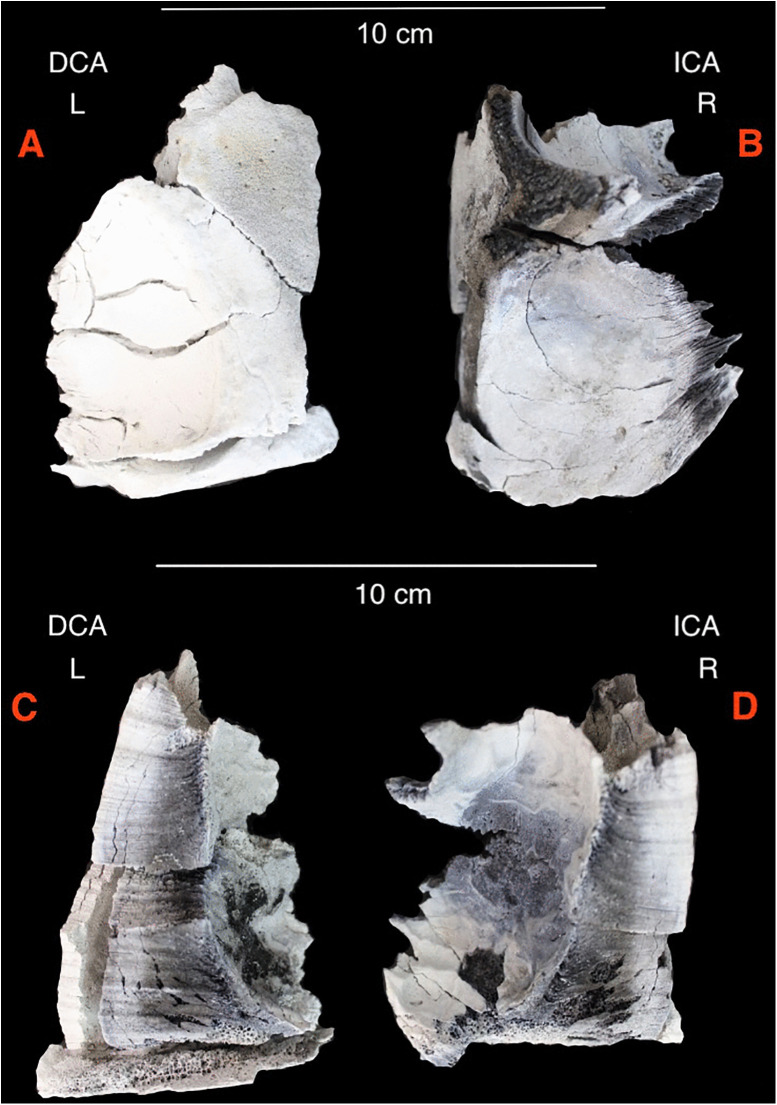
Parietal bones of Head 1 with differences between sides. Outer surface (above: A, B) inner surface (below: C, D).

Preliminary observations indicated that DCA fragments were more calcined (chalky white and brittle), while ICA fragments exhibited darker hues, particularly on inner surfaces. Charred flesh was observed on both heads, limited to ICA fragments, with the exception of internal surfaces of the cranium (Fig 4C).

**Fig 5:**
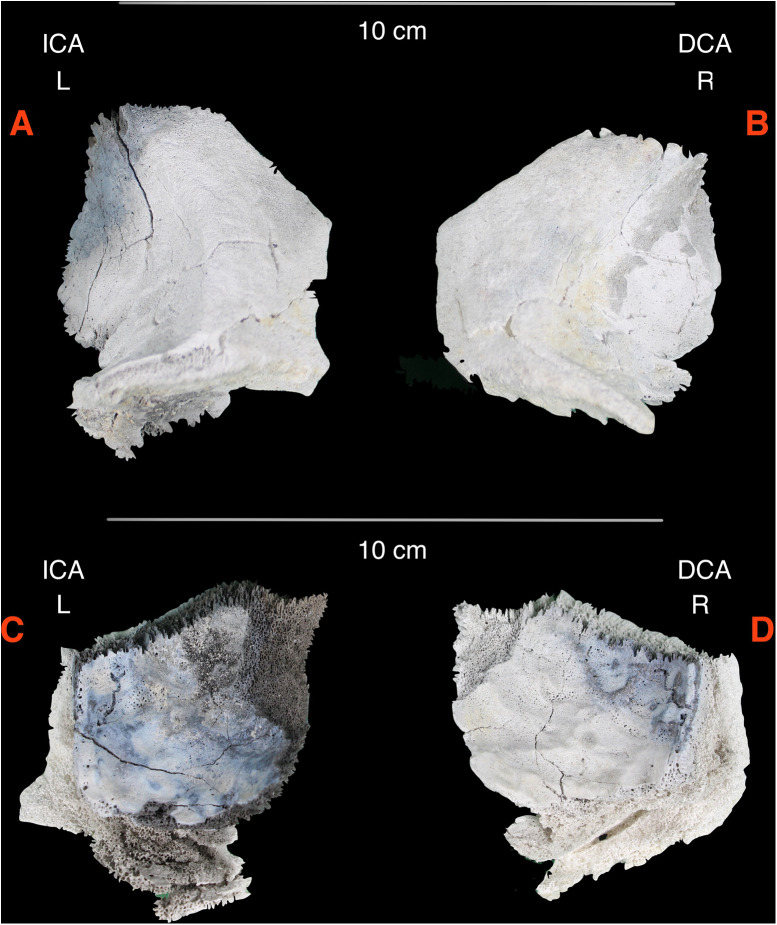
Parietal bones of Head 2 with differences between sides. Outer surface (above: A, B) inner surface (below: C, D).

For Head 1, 74.1% of identifiable fragments larger than 5 mm (700 fragments; 291 g) displayed white as the predominant external colouration, whereas 245 fragments (89 g) corresponded to non-calcined or incompletely calcined remains. In terms of weight, calcined remains represented 76.6% of the analysed assemblage. Ten cranial fragments displayed darker coloration on the internal cortical surface, mainly affecting the left side (DCA; 43 g) and, to a lesser extent, the right side (ICA; 28.7 g) (see S8 File). Colouration patterns appeared to be different between DCA and ICA: DCA fragments were almost entirely white (especially on outer surfaces), while ICA fragments retained darker colouration (Figs 4 and 5). The interior of skulls was consistently darker/bluer than the exterior, probably due to delayed exposure to direct flames. On Head 1, ICA outer surfaces were visibly darker; however, inner colouration was similar across sides (Fig 4C and 4D).

Head 2 displayed less macroscopic variation between DCA and ICA surfaces but retained darker colour on the inner surfaces of ICA (Fig 5C and 5D). Overall, 51% of identifiable fragments larger than 5 mm (876 fragments; 211 g) showed white as the predominant external colouration, whereas 840 fragments (148 g) corresponded to non-calcined or incompletely calcined remains. Calcined remains represented 58.8% of the analysed weight. Four cranial fragments exhibited darker coloration on the internal cortical surface, including two left-sided fragments (parietal and left part of frontal; ICA) representing 21 g, one right parietal fragment (DCA) representing 15 g, and one undetermined neurocranial fragment.

**Fig 6:**
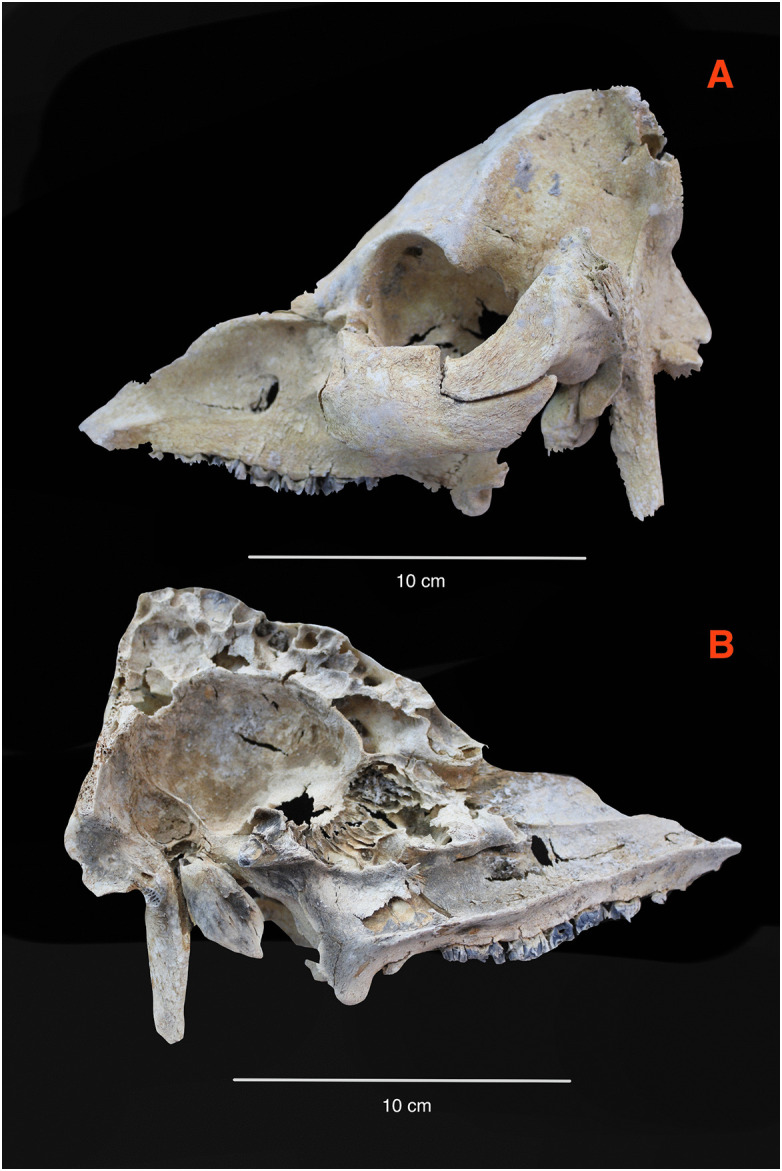
Dry half cranium from Pyre 1 after cremation. A: outer surface (DCA). B: inner surface (ICA).

The dry half cranium displayed a homogeneous calcined colouration in both DCA and ICA. In this case, the outer part as DCA (Fig 6A) and the interior of the skull as the ICA (Fig 6B), showing no clear macroscopic differences in colouration with a warm yellowish value in general (Fig 6). Furthermore, the dry cranium lacked warping and fresh-bone heat-induced fractures.Ch

Overall, Head 1 displayed a higher proportion of calcined remains and greater asymmetry in internal colouration patterns than Head 2, whereas the dry cranium showed the most homogeneous thermal alteration of the three specimens.

### Colorimetry

A total of 153 colorimetric measurements for Head 1, 93 for Head 2 and 24 for the dry cranium, all taken from anatomically identified fragments, were analysed to compare DCA and ICA, and to evaluate how the heterogeneous burning conditions might have influenced these patterns.

- **Head 1 (Pyre 1)**

**Fig 7:**
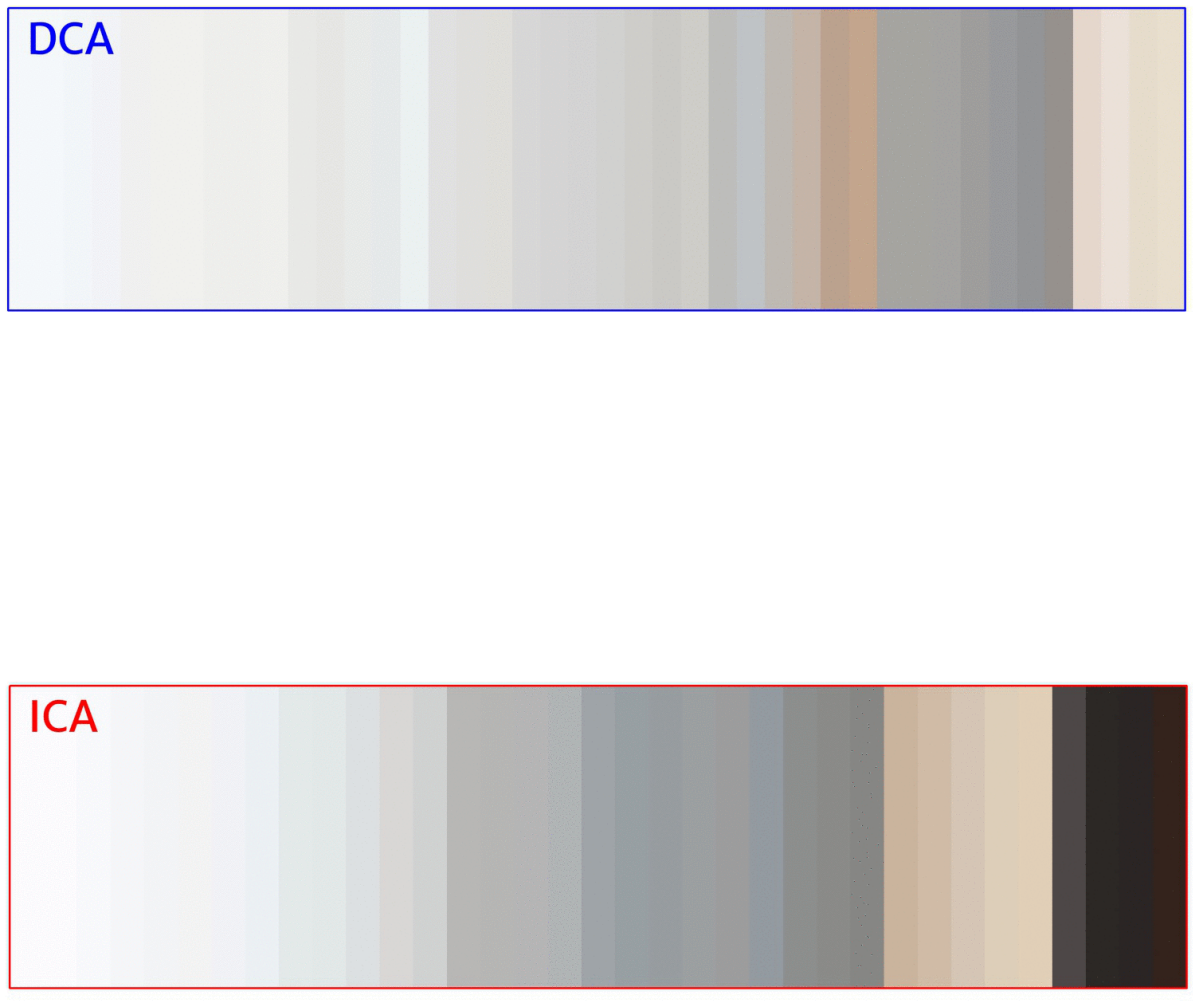
Range of colours obtained by colorimetry analyses on the outer surfaces of DCA and ICA of Head 1.

The fleshed pig head showed significant differences (R2 = 0.06; p-value = 0.004) between DCA and ICA on those bones that were identified with certainty both anatomically and by side, on both outer and inner surfaces. The range of colours when comparing outside surface values from DCA and ICA (present in Fig 9) show only dark colours in the ICA. The inner surfaces of the skulls were generally darker, even in DCA, so they were not included in Fig 7.

- **Head 2 (Pyre 2)**

**Fig 8:**
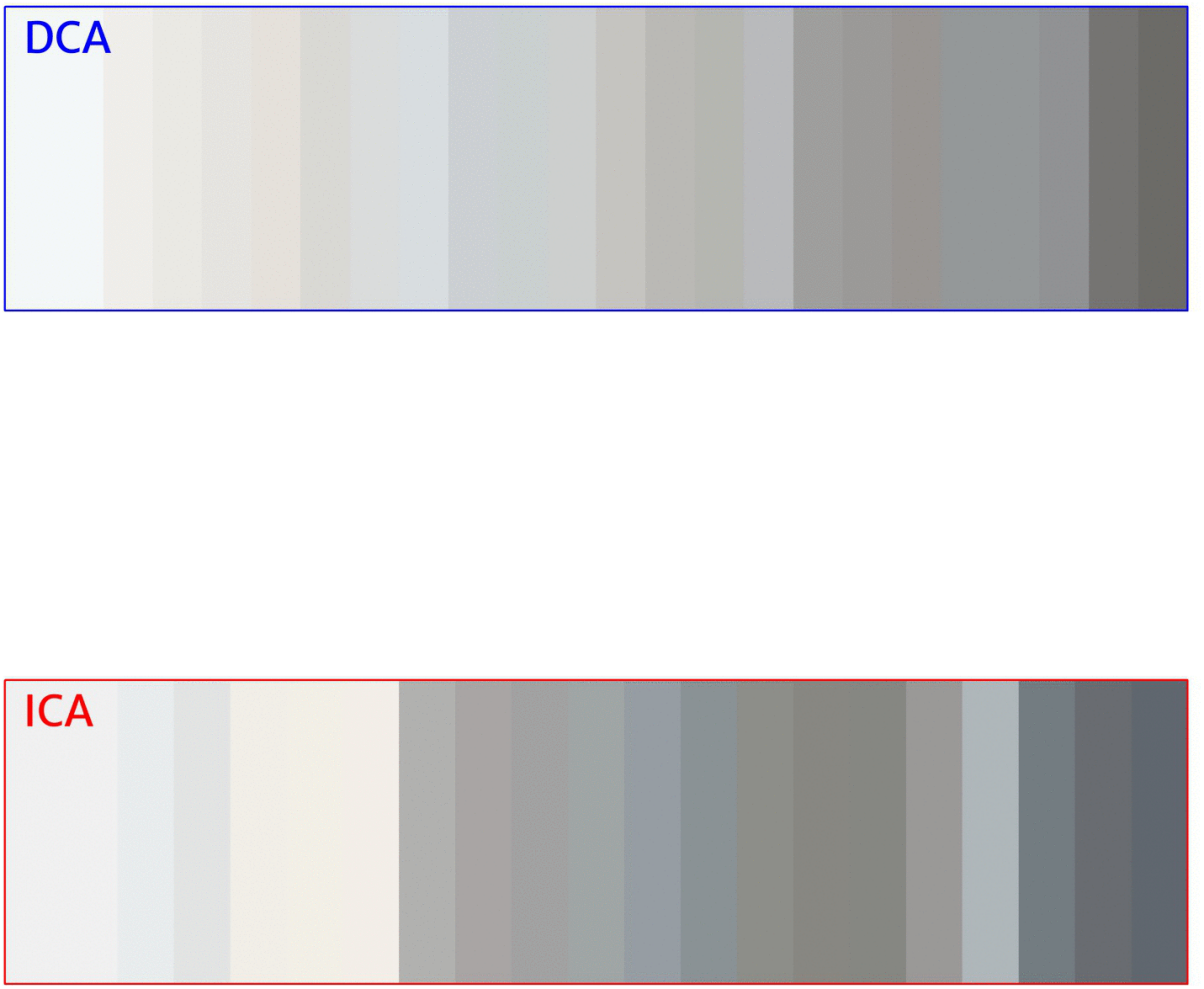
Range of colours obtained by colorimetry analyses on the outer surfaces of DCA and ICA of Head 2.

The fleshed pig head from Pyre 2 also reflected significant differences (R2 = 0.17; p-value = 0.001) between DCA and ICA when comparing both the outer and inner part of the skull. Even so, no differences were appreciated when comparing only the outer surface (R = 0.028 p-value = 0.250) (Fig 8), as observed in the macroscopic features, it displayed less macroscopic variation between DCA and ICA outer surfaces (Fig 5).

- **Dry Cranium vs Fleshed Heads**

**Fig 9:**
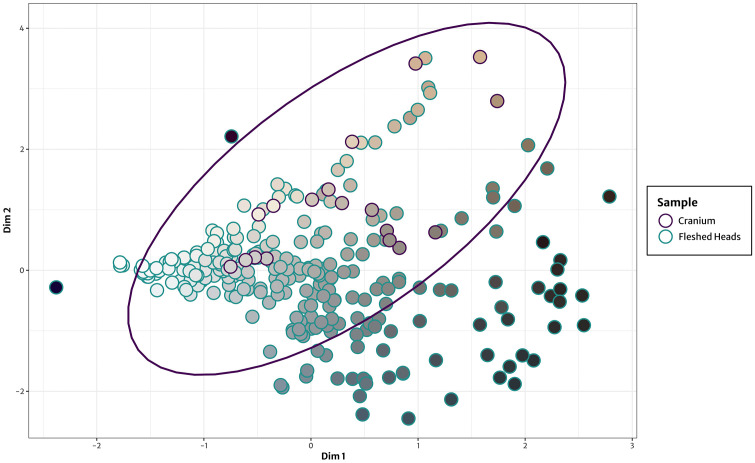
PCOA of L*a*b* values from all areas of both fleshed heads and dry cranium. The purple ellipse marks the values from the dry cranium.

There were visual and graphic differences between all the values registered through the colorimeter on the fleshed heads (Head 1 and 2) and the dry cranium, as visible in Fig 9. However, in the PERMANOVA analysis we did not find significant differences (R2 = 0.01; p-value = 0.075), as there is some chromatic overlap between the samples. Fig 9 shows how the cranium only presented light values and no dark or blueish hues.

- **Head 1 vs Head 2**

Significant differences were also found between DCA on Head 1 and Head 2 (R2 = 0.04; p-value = 0.028), and in ICA (R2 = 0.06; p-value = 0.008). These contrasts may relate to differences in pyre dynamics, particularly the intensity of collapse, although such interpretations cannot be isolated from other uncontrolled variables in this pilot study.

### FTIR-ATR

FTIR-ATR analyses were performed on representative samples from anatomically identified cranial regions (see S3 File: parietals and temporals from both sides of each fleshed head, and a sample from the splanchnocranium of the dry cranium) to investigate chemical alteration under different burning contexts. From the analysed indexes, CN/P, CN2/PO4 and Am/P were not informative in our analyses as they did not yield much quantity in any of the fragments, probably because the FTIR spectrometer was not under vacuum. However, some possible weak cyanamide peaks were observed in the spectra of the two fleshed head (See S7 File) but not in the dry cranium, although their intensity was insufficient to yield measurable values in the CN/P index.

**Fig 10:**
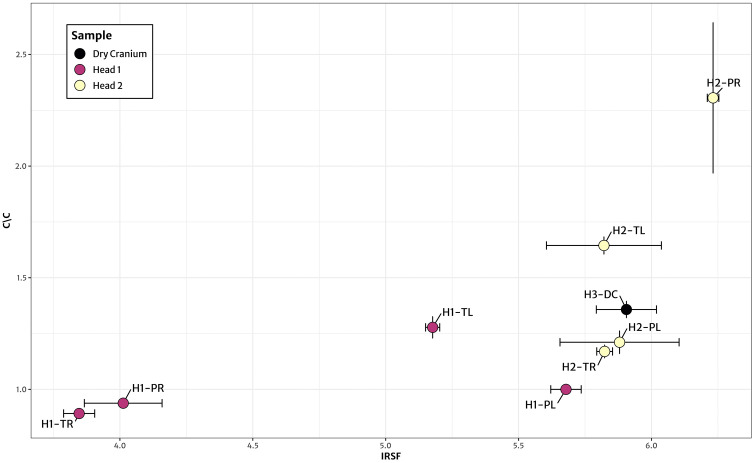
Scatterplot of IRSF versus C/C values according to samples. *The error bars indicate one standard deviation. PL = Left Parietal; TL = Left Temporal; PR = Right Parietal, TR = Right Temporal. DCA corresponds to the left side in Head 1 and the right side in Head 2*.

On the other hand, Infrared Splitting Factor (IRSF), which is informative on the crystallinity of bioapatite [12,15] and carbonyl-to-carbonate ratio (C/C), which evaluates the amount of carbonyls since calcined bones lose this compound completely [15,58,45], showed some interesting differences among individuals (Head 1, Head 2 and Dry Cranium). IRSF presented significantly higher values for Head 2 (Wilcoxon test p-value = 0.03), always above 5.821, while the maximum value for Head 1 was 5.678. IRSF and C/C values in Head 1 showed differences between sides: IRSF was higher in the DCA as expected, as well as a slightly higher C/C content, as seen in Fig 10.

Head 2, however, did not show any side pattern in IRSF or C/C and represents the highest values in C/C in the parietal bone (H2-PR in Fig 10).

**Fig 11:**
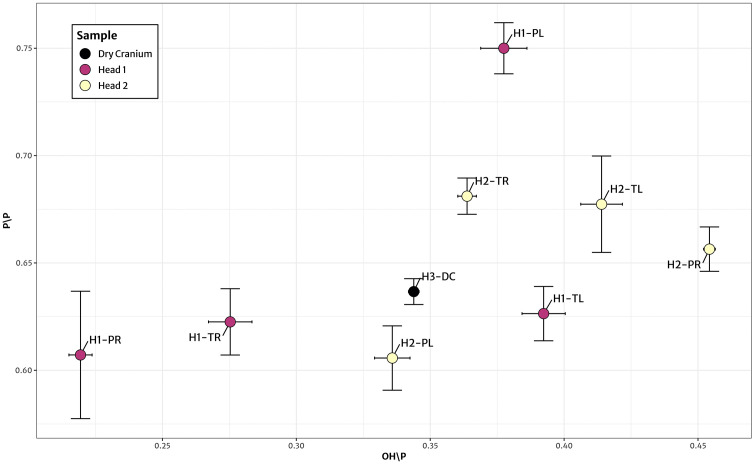
Scatterplot of OH/P to P/P values according to the samples. *The error bars indicate one standard deviation. PL = Left Parietal; TL = Left Temporal; PR = Right Parietal, TR = Right Temporal. DCA corresponds to the left side in Head 1 and the right side in Head 2*.

Phosphate-to-phosphate ratio (P/P), which compares the two absorbance bands of wavelengths associated with phosphates: 600 and 560 cm-1 [12], where the peak at 560 cm ⁻ ¹ becomes more pronounced with higher temperatures [58], and hydroxyl-to-phosphate ratio (OH/P) which evaluates changes in hydroxyl group content in bone bioapatite [15], were also different between individuals. If we compare the OH/P and P/P results in Fig 11, we see a similar trend, where Head 1 displayed differences per side (DCA vs ICA), with higher OH/P and P/P values in the DCA, and Head 2 showed more homogeneity between sides.

Type B carbonate-to-phosphate ratio (C/P), measuring the phosphate peak at 605 cm − 1 and comparing it with the amount of carbonates at 1450 cm-1, where the carbonates being lower the more calcined the sample [12,45], also showed some differences between samples. The highest values in C/P Type B were in the ICA from Head 1 (right side of parietal and temporal bones) (See S3 File), therefore they were probably less exposed to heat, without enough temperature for the loss of carbonates.

## Discussion

The overview of temperatures of both pyres revealed distinct combustion patterns: Pyre 2 underwent greater fluctuations during the first two hours, while Pyre 1 displayed a more abrupt initial temperature rise. A sudden drop in Pyre 1 may reflect a misalignment of the IR pyrometer (see S4 File and descriptions and figures included in S6 File), which may partially explain the lower average temperatures recorded for this pyre. As all readings were measured on the animal samples, these variations represent differences in temperature exposure at the samples level. Given the small scale of this pilot experiment, these thermal differences should be interpreted cautiously. These results might indicate that Head 2 (placed at the bottom) retained heat longer, probably due to pyre structure. Furthermore, Head 1 displayed a higher proportion of identifiable calcined fragments than Head 2 despite the lower average temperatures recorded for Pyre 1, probably because Head 2 exhibited greater fragmentation intensity, with 94% of fragments measuring less than 20 mm in length and numerous small fragments displaying heterogeneous colouration patterns that prevented reliable macroscopic classification. However, darker internal cortical colouration persisted more frequently in Head 1, reinforcing the evidence for heterogeneous heat distribution between the fleshed crania from the two pyres. Colorimetric estimations [44] generally matched pyrometer readings (see S4, S5 and S6 File).The dry cranium yielded anomalously low temperature values in the colorimetric estimations despite being fully calcined, as the FTIR-ATR (S3 File) and macroscopic information (Fig 6) demonstrated, likely due to its orange-hued surface coloration.

As for visual changes in the burning events, the rapid blackening and whitening of the dry cranium in less than 30 minutes is consistent with previous observations (e.g., [34,59]) and highlights the faster combustion of defleshed remains. Chatzikonstantinou et al. [7] also documented high degrees of calcination and fragmentation, attributing the latter to the intensity of the wind. Nevertheless, in our experiments high fragmentation occurred despite minimal wind, suggesting that other factors (e.g., position, carcass weight, fuel amount and distribution) also play an important role. These observations, however, remain hypotheses requiring controlled replication.

Although TEFRA team [7] used larger carcasses and more fuel (1,300 kg), their recorded peak temperatures (801°C) were comparable to ours. Additionally, environmental conditions (e.g., humidity, fuel dryness) likely influenced burn intensity and bone colour. Similarly, Yermán et al. [60] reached temperatures of approaching 1,000 ºC in their one-carcass/one-pyre experiment, comparable with our Pyre 2. However, while they used kerosene and other liquid accelerants, our pyre included artificial fire starters. Their study [60] also discussed the required fuel-to-body-weight ratio for the destruction of organic matter. In our case, both pyres fulfilled their threshold of five times the body weight, with 38.9x in Pyre 1, and 12.9 times in Pyre 2, which may have also contributed to the calcination and fragmentation degrees. However, the influence of fuel ratios cannot be isolated from other uncontrolled variables in just two pilot experiments.

Although seasonal and environmental conditions have been shown to influence combustion dynamics and FTIR-ATR indices [13], their effect could not be independently assessed in the present study due to differences in pyre structure, fuel distribution, and the vertical positioning of the pig crania within the pyres. Head 2 had higher C/C and IRSF values than Head 1, even though it was burned on a cooler and more humid day (6 °C; 78% humidity) and with less wood fuel, suggesting that pyre structure may have enhanced heat retention and efficiency. Given the limited number of experiments and the fact that both pyres were conducted in autumn, the potential effect of seasonality remains tentative.

Regarding mass loss, Jaeger and Johansen [24], reported cremains representing 2.18–3.28% of the original carcass weight in experiments involving complete non-adult pig skeletons. In our case Head 1 accounted for 7.5% and Head 2 for 6% of the original weight, suggesting either lower overall mass loss or higher recovery efficiency, as only skulls were burned. Although these values appear higher, direct comparison should be approached cautiously because only skulls were burned in our experiments, whereas Jaeger and Johansen [24] cremated complete carcasses. The higher percentages observed here may thus reflect differences in anatomical representation and recovery efficiency rather than reduced mass loss alone. Furthermore, our high level of fragmentation could be caused by the delayed collection and exposure to light rainfall [13]. Their three pyres also exhibited temperature fluctuations in the first hour and reached the peak around that same period [24]. Future studies should use thermocouples for more precise thermal profiling and consider comparing recovery strategies, given that manual and delayed collection, as used here to simulate prehistoric contexts, may bias fragmentation metrics.

**Fig 12:**
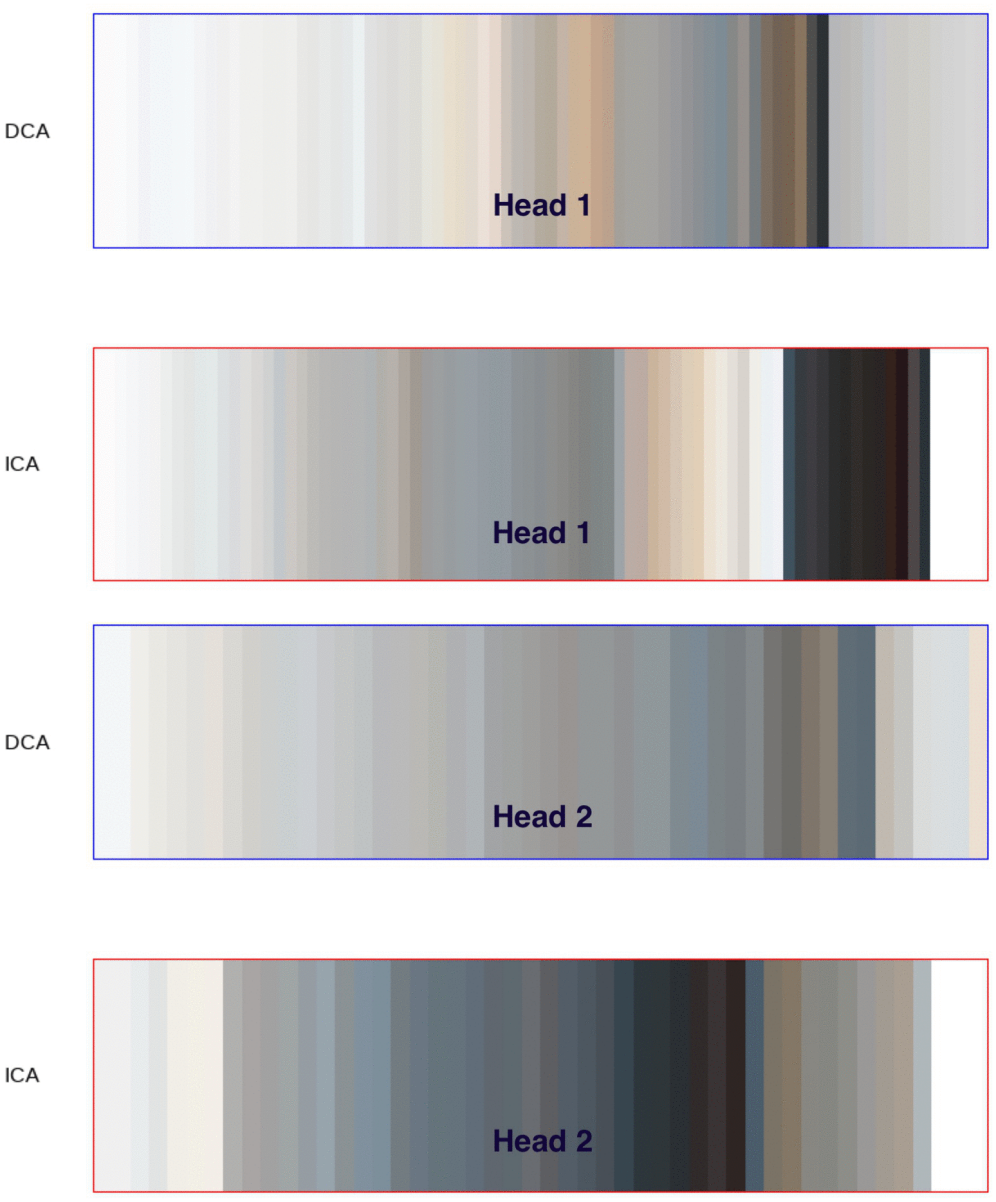
Colour palettes of both heads DCA and ICA including both outer and inner measurements.

Our macroscopic observations revealed preliminary and context-dependent differences in fleshed heads between direct contact areas (DCA) and indirect contact areas (ICA). Colorimetric data showed that DCA surfaces were lighter and more calcined, differing statistically significantly from ICA, even when inner (endocranial) surfaces remained darker due to tissue shielding. These differences were more pronounced in Head 1 (top of Pyre 1), which underwent greater exposure variability due to a subtle pyre collapse. In contrast, Head 2, positioned at the bottom during a pronounced pyre collapse, showed minimal difference between outer ICA and DCA surfaces, although the internal surfaces still displayed asymmetry. Nevertheless, when including the inner surfaces (endocranial), Head 2 had statistically significant differences between sides. Fig 12 displays how the inclusion of the interior values increased the quantity of dark hues on both heads, even in DCA of Head 1, as the interior of the cranium did not reach full calcination. These interesting patterns are, however, exploratory and should be regarded as hypotheses for future testing, given the heterogeneous burning conditions.

These observations also indicate that different vertical placement within the pyre did not generate a consistent lateralised pattern; rather, it might have modified thermal exposure, influencing how clearly lateralised differences could be detected. In this pilot study, the head positioned on top displayed more distinct DCA/ICA contrast. This suggests that vertical position within the pyre may influence the degree to which lateralised burning differences can be macroscopically identified. However, because each vertical position is represented by only one head and the pyres differed in structure and timing, this effect should be interpreted cautiously.

Previous studies have used colour and colorimetry to infer burning temperature [39–41,44,61–64], an approach [44] also applied here just to compare with our infrared measurements (see S5 and S6 File). Colorimetric data have also been used to identify differential heat exposure between anatomical sides, allowing the estimation of body lateral positioning within the pyre in prehistoric human assemblages [29]. Similar patterns of enhanced heating on left-sided elements have been reported in Late Prehistoric Iberia based on the macroscopic degree of burning [6]. Our findings appear compatible with these observations and might be helpful for future archaeological studies, although a larger-scale replication is still necessary.

Fleshed heads exhibited differences across macroscopic, colorimetric and FTIR-ATR analyses. Head 1 showed lateral variation in crystallinity (IRSF), carbonate loss (C/C, OH/P) and phosphate ratios (P/P), with DCA indicating more intense thermal exposure. ICA samples from Head 1 showed higher Type B carbonate-to-phosphate ratios, suggesting less heat-induced carbonate loss. In a previous study [11] lower OH/P values were associated with greater reducing conditions or higher CN/P values. In our samples, the largest OH/P differences were found between DCA and ICA within Head 1. Although some minor spectral variations were visible in the cyanamide wavenumber region (S7 File) it did not yield sufficient signal for CN/P quantification. Our lower File OH/P values in the less burned area are similar to the values of the less burnt shoed foot on their study [11].

On the other hand, while Head 2 also displayed side differences in colorimetry, these were not mirrored in FTIR-ATR indices. This discrepancy may reflect the more subtle macroscopic differences observed between sides in Head 2 compared with Head 1, possibly due to a more homogeneous heat distribution related to pyre structure and vertical positioning. Even if the reducing conditions were accentuated because of the vertical position of this head, it was not evident in either the OH/P or CN/P values.

Rather than compromising the usefulness of FTIR-ATR in cremation studies, the limited resolution in this setup, suggest that the method may be more sensitive to pronounced or sustained thermal differences than to relatively subtle lateralised variation in heat exposure and that further interpretation require controlled future testing. However, the side-specific trends observed in Head 1 may support the potential value of FTIR-ATR for investigating indicators of body positioning on pyres under certain conditions.

Dry and fleshed elements also behaved differently, affecting the potential identification of DCA vs ICA in dry remains. The dry cranium displayed a uniform yellow-orange colouration, very low fragmentation, no warping, and lacked the heat-induced fractures typically associated with fleshed (“fresh”) bone, such as thumbnail fractures, displaying just some delamination (see S8 File), all of which were supported by colorimetric and mass-loss data. Although no statistical differences were found when all the colorimetric values were considered, unlike in other studies [40], in both cases, dry bones tended to display warmer orange hues. However, the aforementioned darker internal colouration could potentially serve as an additional proxy for pre-burning condition, such as presence of soft tissue or cranium completeness. All these colorimetry results, which seem promising for exploring pre-burning states in archaeological contexts, need to be further validated with future controlled experiments, as the experiment used just three specimens and their thermal exposure differed in timing and context, these contrasts cannot yet be attributed to pre-burning state alone. Nevertheless, these findings indicate that, under the conditions of this pilot study, FTIR-ATR and colorimetry (beyond differences between inner and outer surfaces) did not consistently distinguish between dry and fleshed remains. In contrast, macroscopic observations, particularly fragmentation patterns, colour homogeneity, warping, and the absence of fresh-bone heat-induced fractures in the dry cranium, provided clearer differentiation between pre-burning conditions. These results therefore suggest that macroscopic evaluation may represent a valuable and accessible proxy for assessing pre-burning conditions in archaeological and forensic contexts where destructive analyses of human remains are restricted or not feasible.

As a synthesis of the multiproxy results, Head 1, positioned on top of the pyre, exhibited clearer lateralised differences between DCA and ICA, a higher proportion of identifiable calcined fragments, and side-specific variation in several FTIR-ATR indices, suggesting more heterogeneous and laterally differentiated heat exposure. In contrast, Head 2, positioned at the bottom of the pyre, showed greater fragmentation, higher average temperatures, and more homogeneous FTIR-ATR values, despite still retaining some asymmetry in colorimetric and macroscopic patterns. The dry cranium differed greatly from both fleshed heads, with low fragmentation, homogeneous colouration, and absence of typical “fresh-bone” heat-induced fractures. Together, these results suggest that pyre structure, vertical positioning, and pre-burning condition influenced the expression and detectability of lateralised burning patterns in this pilot study.

FTIR-ATR, thus, showed minimal chemical variation, indicating that, in this particular dataset, the measurements were less sensitive to differences in moisture or collagen content. FTIR-ATR under vacuum could improve the reconstruction of pre-burning condition, for example with more accurate CN/P values that could amplify the subtle peaks observed in S7 File, as the presence of ammonia is related to reducing atmospheres and the amount of organic matter [11]. This signal may also be influenced by the presence of shrouds or footwear [11,12]. Hence, such reducing conditions could potentially result from the shielding effect of thicker soft tissues. However, comparisons between the fleshed heads and the dry cranium must be interpreted cautiously, as the latter was introduced later into the pyre. These differences in timing and thermal exposure may also have influenced the resulting FTIR-ATR values, colouration patterns and fragmentation levels.

The dry cranium showed the second highest IRSF value, while C/C values fell between those of Head 1 and Head 2 (Fig 10), suggesting that the original values, that could be illustrative of the post-mortem interval, were probably obscured by burning effects [65]. In the experiment of Zazzo et al. [66], much higher IRSF values were also observed, likely due to recrystallization of archaeological bones. Overall, our IRSF values were also higher than those reported for calcined samples in the cremation experiments of Snoeck et al. [58], except for the ICA areas of Head 1, which also showed comparable C/P values to the charred samples published by the same authors [58].

Overall, the results of this study suggest that the identification of differential burning patterns might be influenced by the discussed factors: (1) the pre-burning state of the remains (fleshed vs. dry), (2) their vertical position within the pyre, which acted as a modifier of thermal exposure rather than a generator of a consistent pattern, and (3) pyre dynamics such as fuel distribution, collapse processes and environmental conditions. These variables influence the expression of lateralised burning patterns and should be controlled in future replicated experiments to refine and standardise the methodology.

Our preliminary results suggest that lateral differences in thermal exposure may arise in certain contexts, indicating that this multiproxy approach warrants further testing for remains in lateral, prone or supine positions, with adjusted DCA/ICA mapping. In fact, prior forensic studies also reported asymmetric burning on soft tissues depending on position [3, 28–34, for instance, with the absence or diminishment of a pugilistic attitude in side-lying or prone positions [30], or the limited exposure of skin surfaces in direct contact with the ground to flame impact [32]. Other studies have highlighted how these thermal asymmetries can indicate displacement of the body from its original position [32,34,35].

### Limitations of the pilot study

One of the limitations of this study is the extrapolation of results from animal carcasses to human cases. Although the predictable burning patterns observed in human head tissues during thermal destruction [33] are not fully comparable to the pig soft tissues, the positioning of heads and the shielding effect of soft tissues from the direct flame contact have been demonstrated in several publications [3,32,35,36]. Furthermore, most experimental approaches have used pig carcasses [7,11,13–15,18,25,26,37,40,41,60,65–71] due to the similarities to human tissues [38,41].

A second limitation lies in the exclusive use of heads rather than complete carcasses, and the limited number of experiments. In forensic cases, complete bodies have shown additional differential burning patterns influenced by anatomical placement, soft tissues and body fluids [3,33,34]. Therefore, the limited number of experimental pyres implies that this study should be regarded as a “benchmark experiment” [39] and its conclusions applied cautiously in archaeological contexts, and even more so in forensic contexts.

Additional limitations include pyre heterogeneity, environmental variation between experiments, different timing of introduction of the dry cranium, variation in soft-tissue removal, and the outdoor recovery process, all of which may have contributed to variation in the results. These factors highlight the need for controlled, replicated studies to test the hypotheses proposed here.

However, even with the exposed limitations, this study illustrates the feasibility of using multiproxy approaches to explore vertical and lateral body placement in experimental studies, as both appear, in this pilot study, to influence burning outcomes, and the importance of also testing burning patterns in cranial structures [33]. However, future work should include additional experiments incorporating more anatomical regions, varied decomposition stages, and controlled pyre configurations to refine interpretive frameworks and validate our methodology.

## Conclusion

This pilot study provides preliminary evidence for the the use of multiproxy methodologies for exploring how body positioning on left or right-side may influence burning outcomes, particularly calcination in DCA and the presence of darker values in ICA. Both areas could be identified through macroscopic features, colorimetry and in some cases FTIR-ATR values in our limited number of experiments, but these results should be interpreted within the limits of a pilot experimental framework. The preliminary results also showed that the lateralised pattern can be affected by the other variables under study:

1. Fleshed and dry remains behaved differently under cremation conditions: fleshed heads showed greater fragmentation, mass loss, wider colour variability, and fracture patterns, whereas dry crania exhibited less fragmentation and more uniform burning effects, without DCA/ICA differences.
2. In fleshed heads macroscopic observations and colorimetry allowed the identification of DCA and ICA, FTIR-ATR supported these observations in a limited number of cases, but overall neither FTIR-ATR nor colorimetry consistently distinguished between fleshed and dry remains.
3. Vertical placement in the pyre and collapse processes affected the clearness of differentiation of DCA and ICA in FTIR-ATR results and, to a lesser extent, colorimetry, with greater differentiation in the head placed on top of the pyre than in the head placed at the bottom.
4. Decomposition state, biological variation, pyre structure and fire dynamics introduce substantial heterogeneity into cremation outcomes. This inherent variability complicates interpretations of burning conditions and lateralised body positioning and underscores the need for cautious, context-dependent interpretation of cremated remains using multiproxy approaches.

Given the heterogeneity observed, future work should also test different pyre types under homogeneous conditions. In summary, while lateralised burning patterns were detectable through macroscopic, colorimetric and FTIR-ATR analyses, these findings should be regarded as preliminary indicators of the potential of this multiproxy methodology rather than definitive patterns.

## Supporting information

S1 File Standardised form for experimental pyres. (PDF)

S2 File 3D model of Pyre 1. All objects must be opened within the same file. (ZIP)

S3 File FTIR-ATR Results. (XLSX)

S4 File Temperature measurements. (XLSX)

S5 File Colorimetric measurements and estimated temperatures. (XLSX)

S6 File Detailed experimental pyre changes and temperatures overview. (DOCX)

S7 File FTIR spectra of representative DCA samples from Head 1 (H1), Head 2 (H2), and the dry cranium (H3). Peak descriptions were added following Snoeck et al. [15].
(PNG)

S8 File Osteological analysis and macroscopic observations. (XLSX)
