# Correction: Assessing body position through experimental cremation: A pilot study using colorimetry and FTIR-ATR analyses

**DOI:** 10.1371/journal.pone.0353659

**Published:** 2026-07-10

**Authors:** Paula Becerra Fuello, Javier Lescure, Aaron Lackinger, María Sedeño Ráez, Jesús Gámiz Caro, Gonzalo Aranda Jiménez

The author initials appear incorrectly in the citation. The correct citation is: Becerra Fuello P, Lescure J, Lackinger A, Sedeño Ráez M, Gámiz Caro J, Aranda Jiménez, G. (2026) Assessing body position through experimental cremation: A pilot study using colorimetry and FTIR-ATR analyses. PLoS One 21(6): e0351767. doi.org/10.1371/journal.pone.0351767

The ORCID iD is missing for the sixth author. Please see Gonzalo Aranda Jiménez’s ORCID iD here: 0000-0003-1925-0221 (https://orcid.org/0000-0003-1925-0221).
