# Supplementary material for: Assessing body position through experimental cremation: A pilot study using colorimetry and FTIR-ATR analyses
Source: PLoS One. 2026 Jun 15;21(6):e0351767. doi: 10.1371/journal.pone.0351767 (PMC13268179; doi:10.1371/journal.pone.0351767)
Supplement: S1 File — (PDF) [file pone.0351767.s001.pdf]

EXPERIMENTAL PYRE FORM

RESEARCHER NAME:

GEA

UNIVERSIDAD DE GRANADA

GENERAL DATA

Experiment Number: ______________

Type of pyre: ______________

Location: ______________

Time: ______________

ENVIRONMENTAL DATA

Humidity: ______________

Temperature: ______________

Wind: ______________

MATERIAL

Fuel Type: ______________

Fuel Weight: ______________

Sample type: ______________

Sample Weight ______________

DIMENSIONS

Length x Width ______________

Height: ______________

TEMPERATURE RECORDING

Comments/ Changes	Time	ºC	PHOTO
IGNITION			☐
			☐
			☐
			☐
			☐
			☐
			☐
			☐
			☐
			☐
			☐

COMMENTS ON PHOTOS

____

____

____

COMMENTS ON CHANGES

Collapse, charring of flesh, calcination, etc.

____

____

____

COMMENTS ON SENSORY EXPERIENCE

Smell, fumes, brightness, heat, sounds

____

____

____

____

EXPERIMENTAL PYRE FORM

RESEARCHER NAME:

GEA
UNIVERSIDAD DE GRANADA

ADDITIONAL OBSERVATIONS

END OF THE EXPERIMENT

Final appearance

DIMENSIONS

Pyre debris: ______ Weight: ______

Cremains: ______ Weight: ______

RECOVERY

Charcoals, coals	☐	Anatomical connection	☐
Bone visibility	☐	High fragmentation	☐
Calcined remains	☐	Red-hot/white-hot bones	☐
Charred flesh	☐	Ash covering	☐

PYRE EXTINQUISHING

Water ☐ Liters: ______ No water/Once cold ☐ Date ______

While still hot ☐ Temperature: ______ Time: ______

SKETCH
