# Supplementary figures and images for: Assessing body position through experimental cremation: A pilot study using colorimetry and FTIR-ATR analyses
Source: PLoS One. 2026 Jun 15;21(6):e0351767. doi: 10.1371/journal.pone.0351767 (PMC13268179; doi:10.1371/journal.pone.0351767)

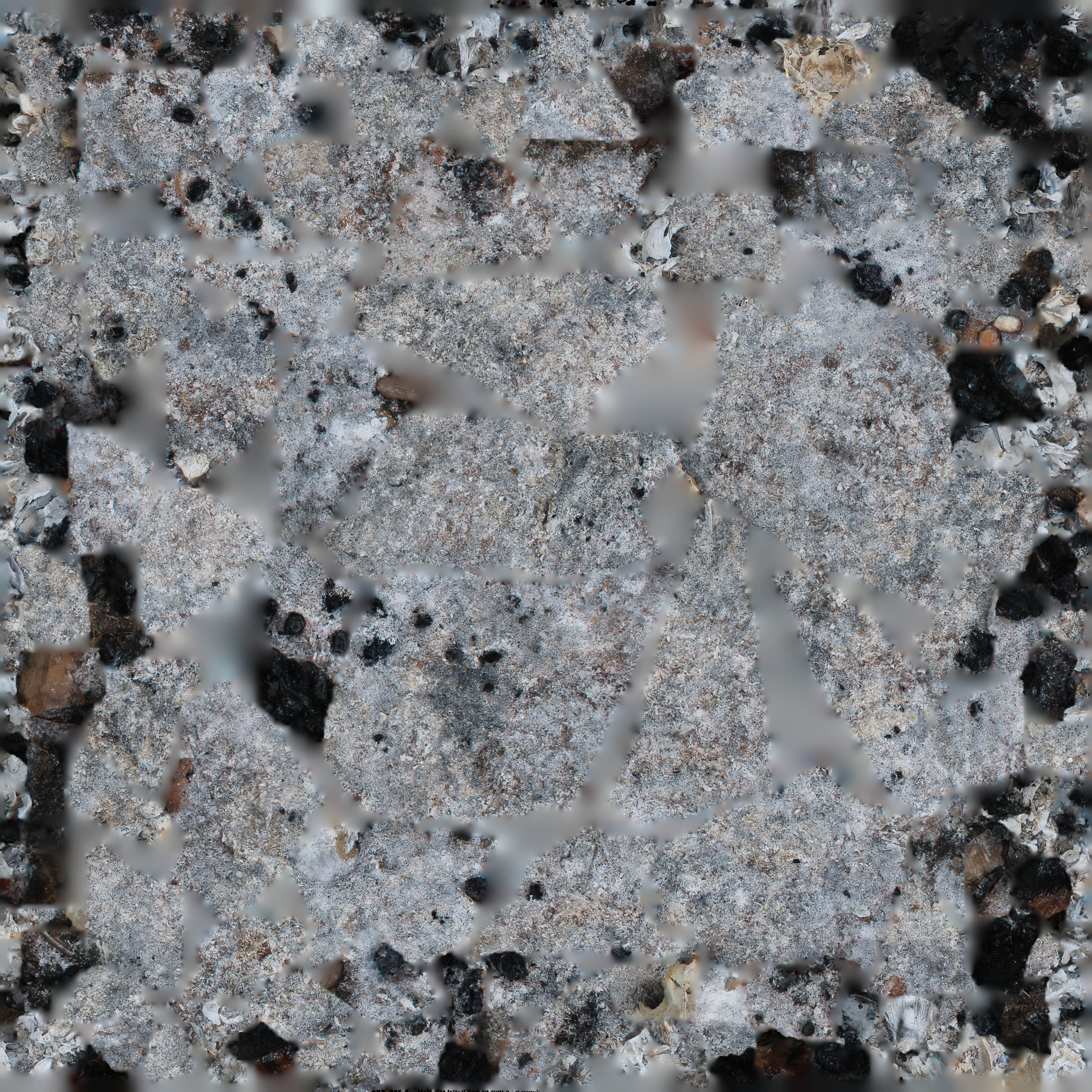

Supplement: S2 File — (ZIP) [file pone.0351767.s002.zip › PIRA_PAULA_MODELO_3D_alta/PIRA_DETALLE_1.1001.jpg]

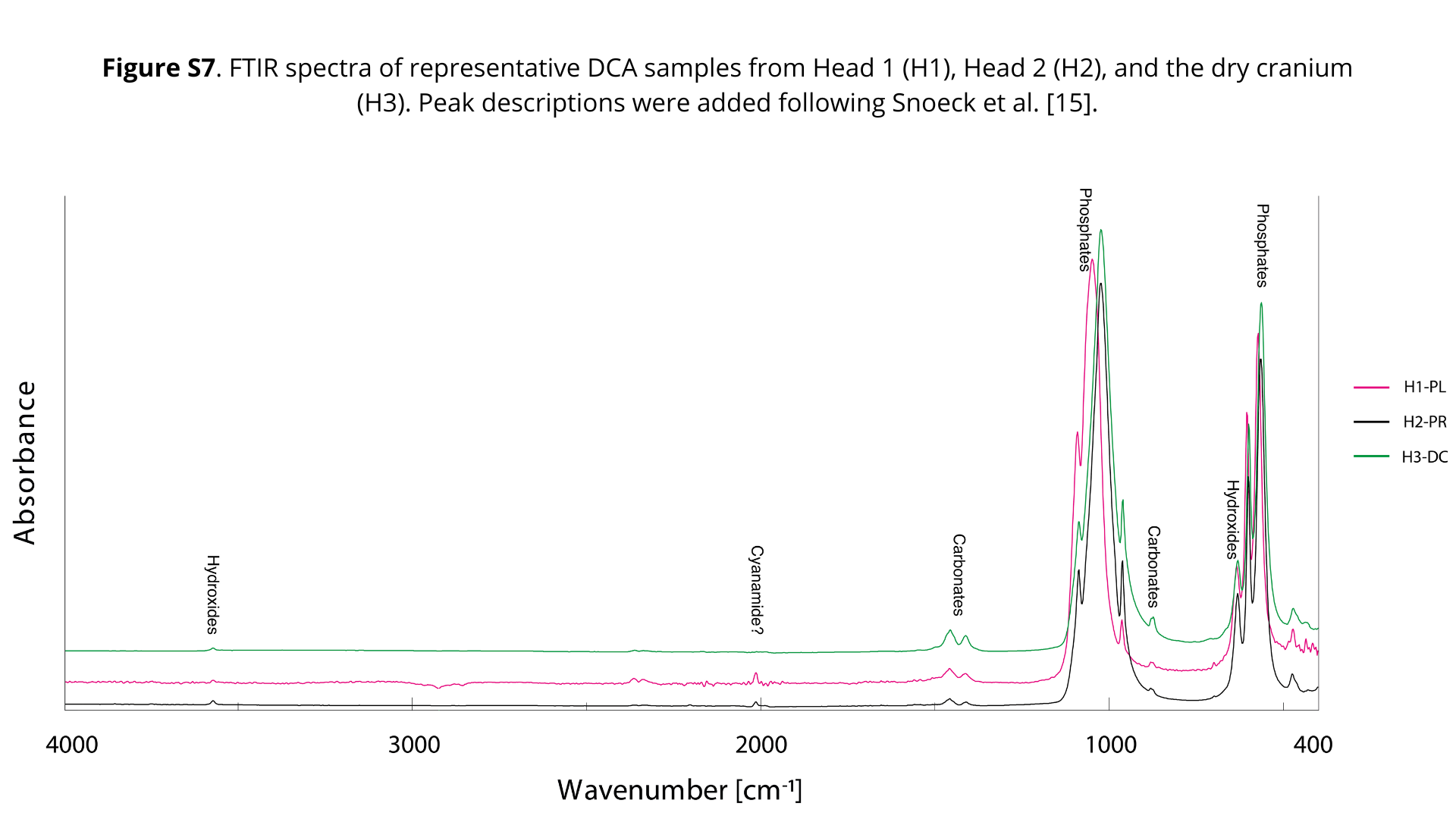

Supplement: S7 File — Peak descriptions were added following Snoeck et al. [15]. (PNG) [file pone.0351767.s007.png]
