# Supplementary material for: Assessing body position through experimental cremation: A pilot study using colorimetry and FTIR-ATR analyses
Source: PLoS One. 2026 Jun 15;21(6):e0351767. doi: 10.1371/journal.pone.0351767 (PMC13268179; doi:10.1371/journal.pone.0351767)
Supplement: S6 File — (DOCX) [file pone.0351767.s006.docx]

**Supporting Information 6**

Detailed experimental pyre changes and temperatures overview:

**PYRE 1:**

Ignition began at 08:08 from different points around the pyre, including both the base and middle layers. The fleshed pig head (5.05kg) was placed on its left side in direct contact with the wood. At 08:52, half a dry cranium (120g pre-cremation weight) was added. The dry cranium was added later because given the lack of flesh, calcination could occur faster than with the fleshed head, as it happens in thinner soft tissues of the skull in forensic cases [e.g. 31]. In fact, it quickly turned black and later white and calcined (with a chalky and brittle appearance), just 30 minutes after its introduction. The wind shifted in direction to come from the southeast at 3.6 km/h when the cranium was introduced. The experiment lasted 8 hours, ending at 16:15.

Temperature Overview:

Pyre 1: During the eight-hour cremation process, the average temperature was 390 °C for the fleshed head and 345 °C for the dry cranium. The maximum temperature (870 °C) was recorded on the dry cranium half around 30 minutes after placement. Figure 4 illustrates the temperature variations recorded with the IR pyrometer.

At 8:12, around 5 minutes after ignition, we added more logs on the western side of the pyre (included in the total weight), as that area ignited more quickly. At 8:20, the pig head shifted slightly towards the pyre centre; probably affecting the temperature reading (mean 400°C max and 300°C min), which is interpreted as an outlier, probably due to a misalignment in the pyrometer. By 08:52, most of the exposed flesh was charred, with the complete loss of ear cartilage. Flames reached over 1.5m in height, i.e. when the dry cranium was added. The dry cranium quickly blackened and became calcined less than 30 minutes after its placement, at 9:25, just as the pyre reached it maximum temperature. At 09:42, the maxilla was fully exposed and the palate had detached. At 10:26, the pyre partially collapsed towards the centre, losing its original height. At 12:00 the pyre was reduced to ashes and embers. By the end of the fire event, at 16:15, some of the ashes were still at 230ºC residual temperature.


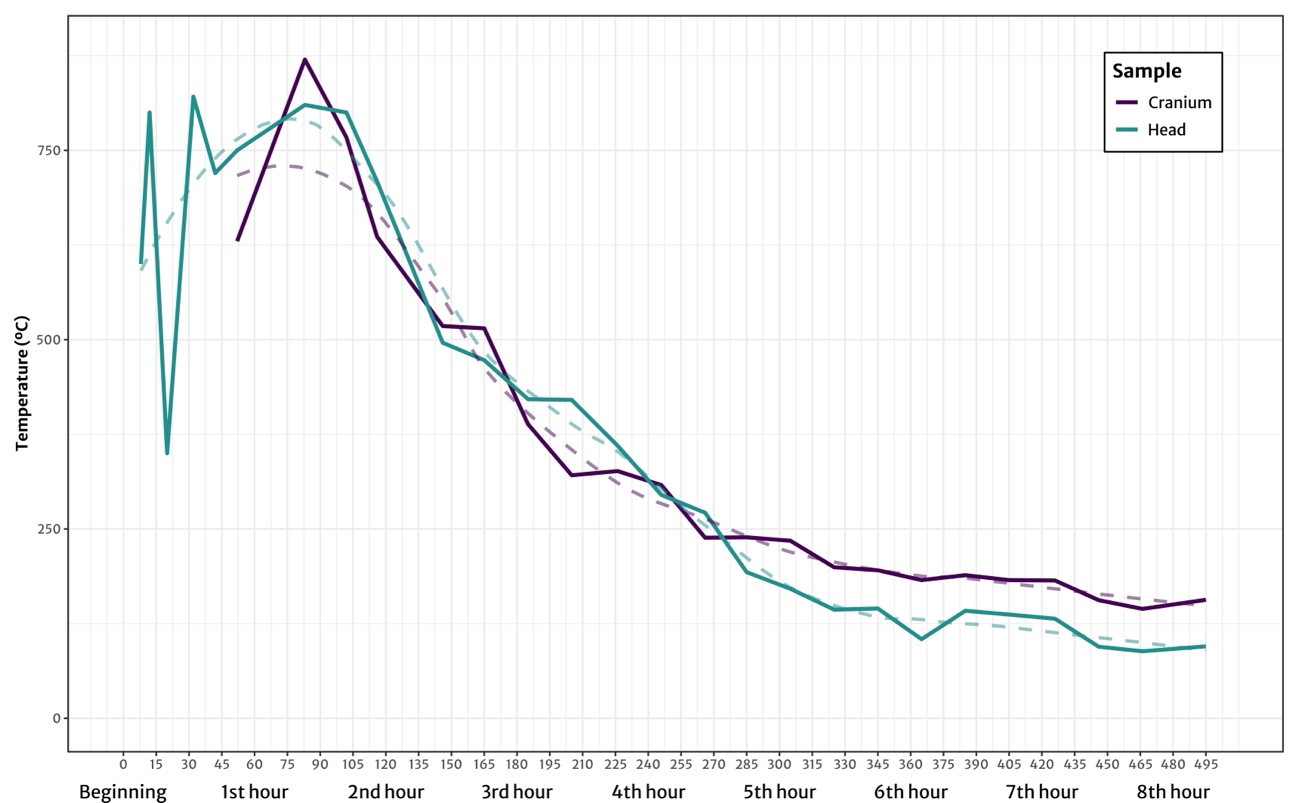


Average temperatures measured via IR Pyrometer on the fleshed head and dry cranium from Pyre 1. Smooth curves (dashed) were fitted by Local Polynomial Regression (loess).


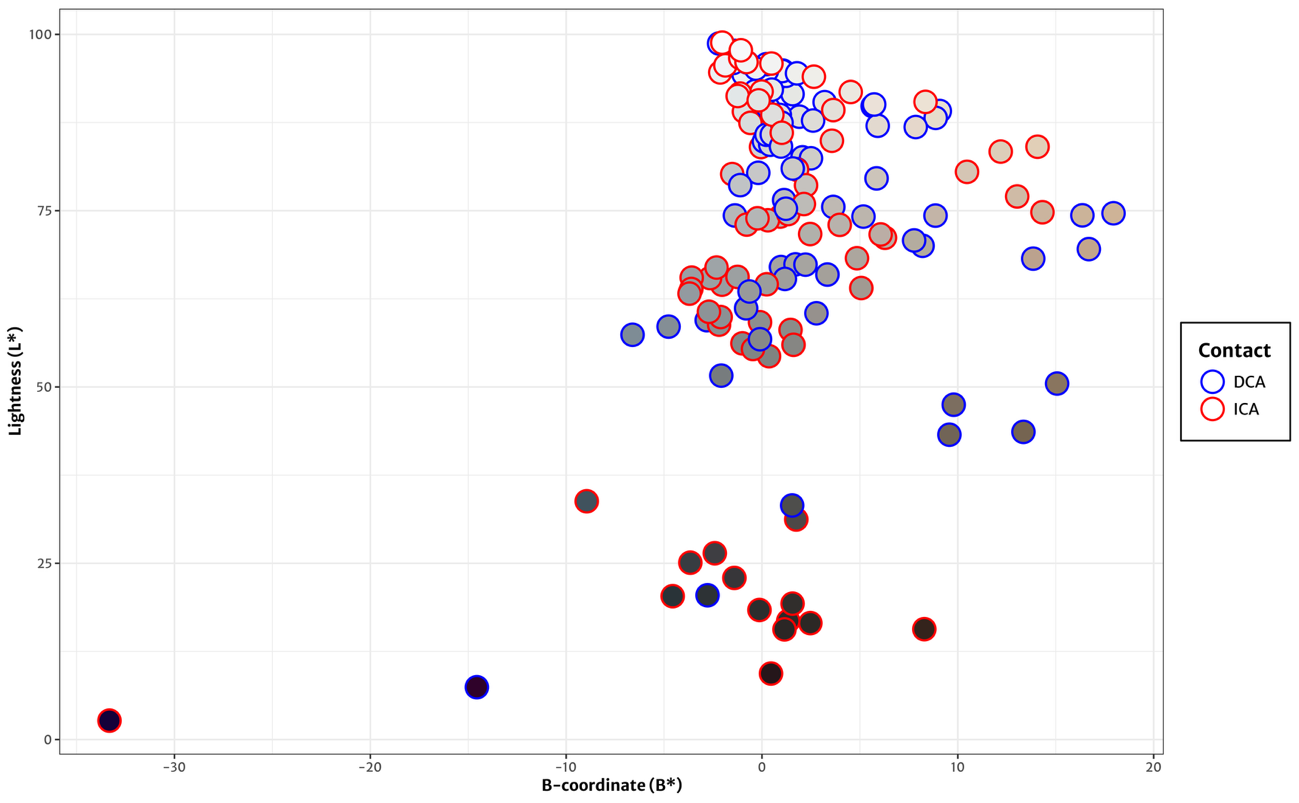


L* and b* coordinates for Head 1 that reflect differences in potential temperature reached based in Krap et al. [51] Figure 1, where most of our calcined samples (located at the upper area of the plot) range and most of the non-calcined falling in the 350-500 range (and mostly belonging to ICA).

**Pyre 2:**

The experiment began at 9:48 under the ambient conditions described in the main text. The collapse and combustion of the structure occurred more rapidly and intensely than with Pyre 1. Just 30 minutes after ignition, at 10:15, the soft tissues were charred and some of the bones exposed. At 10:45 the top of the pyre collapsed over the head. At 12:00 most of the logs were embers and only the outer ring had not properly burned. This experiment was shorter as it was concluded once all the logs had fully turned to ash (except those on the outer ring that were never completely burned). Therefore, the temperature measurements ended at 14:25. Bone remains were also collected three days later, on December 16.

Temperature overview:

At 9:48 the pyre was finally ignited. The experiment lasted until the bones were completely exposed and calcined and most of the fuel had turned to ash. The average temperature was 644.6ºC, higher than the previous experiment. However, this is because temperature recording stopped after the calcination of the bones and the complete loss of logs, and because the maximum temperature of 953ºC was measured on charred flesh. That temperature was reached on the head when most of the visible flesh had been charred at 10:15, just 35 minutes after ignition. At 10:30 some parts of the skull were visibly calcined. Fifteen minutes later, at 10:45, the pyre collapsed over the cranium. At 11:30 most of the pyre had been consumed and it was just 30 cm high and there was visible fragmentation of the cranium. At 14:25 we concluded the measurements as most of the ignited logs had been reduced to ashes. All the described events can be evaluated in the following figure.


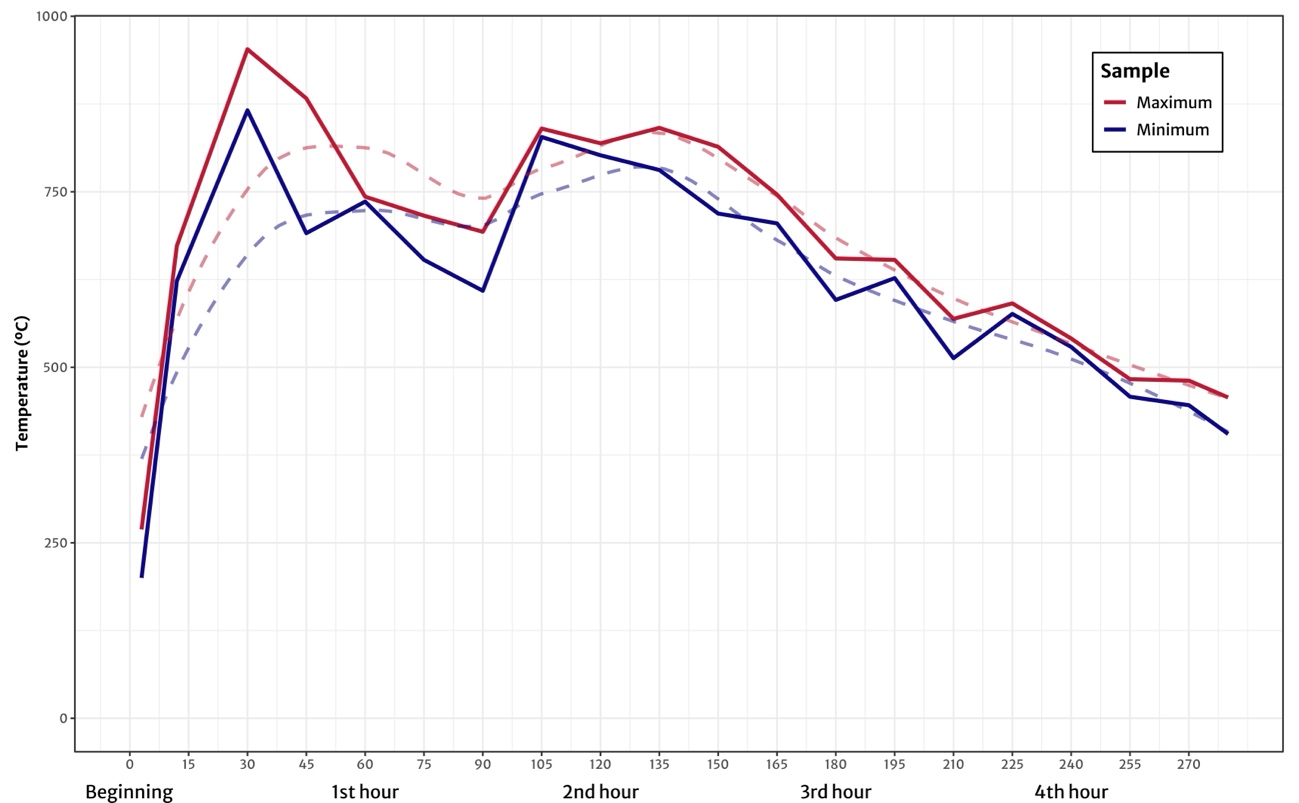


Average temperatures measured via IR Pyrometer on the fleshed head from Pyre 2 with maximum and minimum. Smooth curves (dashed) were fitted by Local Polynomial Regression (loess).


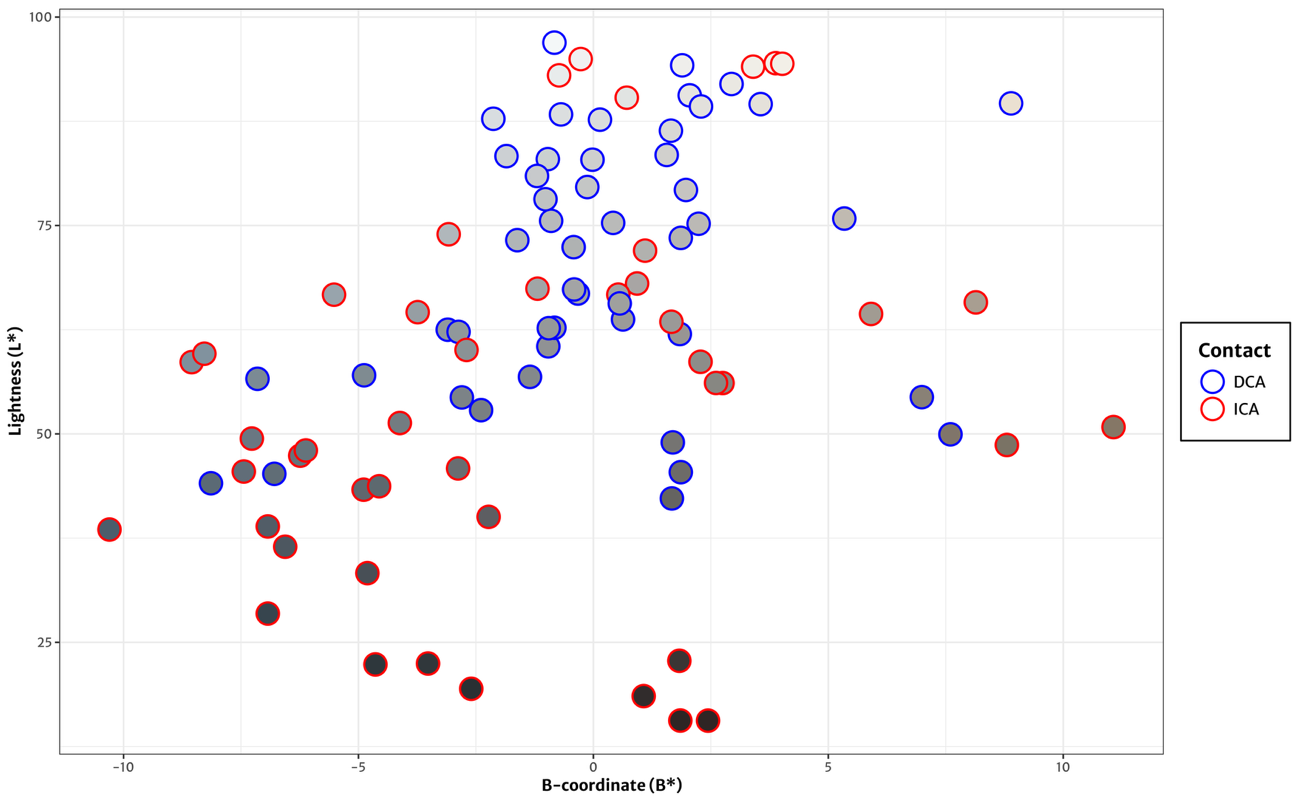


L* and b* coordinates for Head 2 reflect differences in the estimated reached temperatures based on Krap et al. [51] Figure 1, where most of our calcined samples (located at the upper area of the plot and mostly belonging to DCA) would fall within the 700-900 ºC range, and most of the non-calcined ones within the 350-500 range. However, the latter generally exhibited lower b* values than those artificially heated in [51], thus displaying therefore a more bluish hue [39] than predicted by the model.
